# Technological solutions for an effective health surveillance system for road traffic crashes in Burkina Faso

**DOI:** 10.1080/16549716.2017.1295698

**Published:** 2017-06-02

**Authors:** Emmanuel Bonnet, Aude Nikiéma, Zoumana Traoré, Salifou Sidbega, Valéry Ridde

**Affiliations:** ^a^ UMI Résiliences 236, French National Research Institute for Sustainable Development (IRD), Bondy, France; ^b^ CNRST, Institut des Sciences des Sociétés (INSS), Ouagadougou, Burkina Faso; ^c^ Africasys, Paris, France; ^d^ Département de Géographie, Université de Ouagadougou, Ouagadougou, Burkina Faso; ^e^ University of Montreal School of Public Health (ESPUM), Montreal, Canada; ^f^ University of Montreal Public Health Research Institute (IRSPUM), Montreal, Canada

**Keywords:** Road safety, public health, trauma, injury, system

## Abstract

**Background**: In the early 2000s, electronic surveillance systems began to be developed to collect and transmit data on infectious diseases in low-income countries (LICs) in real-time using mobile technologies. Such surveillance systems, however, are still very rare in Africa. Among the non-infectious epidemics to be surveilled are road traffic injuries, which constitute major health events and are the fifth leading cause of mortality in Africa. This situation also prevails in Burkina Faso, whose capital city, Ouagadougou, is much afflicted by this burden. There is no surveillance system, but there have been occasional surveys, and media reports of fatal crashes are numerous and increasing in frequency.

**Objective**: The objective of this article is to present the methodology and implementation of, and quality of results produced by, a prototype of a road traffic crash and trauma surveillance system in the city of Ouagadougou.

**Methods**: A surveillance system was deployed in partnership with the National Police over a six-month period, from February to July 2015, across the entire city of Ouagadougou. Data were collected by all seven units of the city’s National Police road crash intervention service. They were equipped with geotracers that geolocalized the crash sites and sent their positions by SMS (short message service) to a surveillance platform developed using the open-source tool Ushahidi. Descriptive statistical analyses and spatial analyses (kernel density) were subsequently performed on the data collected.

**Results**: The process of data collection by police officers functioned well. Researchers were able to validate the data collection on road crashes by comparing the number of entries in the platform against the number of reports completed by the crash intervention teams. In total, 873 crash scenes were recorded over 3 months. The system was accessible on the Internet for open consultation of the map of crash sites. Crash-concentration analyses were produced that identified ‘hot spots’ in the city. Nearly 80% of crashes involved two-wheeled vehicles. Crashes were more numerous at night and during rush hours. They occurred primarily at intersections with traffic lights. With regard to health impacts, half of the injured were under the age of 29 years, and 6 persons were killed.

**Conclusions**: This pilot study demonstrated the feasibility of developing simple surveillance systems, based on mHealth, in LICs.

## Background

In the early 2000s, electronic surveillance systems began to be developed to collect and transmit data on infectious diseases in low-income countries (LICs) in real-time using mobile technologies. Information and communication technologies (ICTs) were developed that offered sophisticated computing and communications capabilities from remote settings [1]. These ICTs enabled the development of public health surveillance systems [2] to record health events affecting populations, with the ultimate aim of supporting the development of health interventions [2].

Such surveillance systems are still very rare in Africa, especially in LICs [3], despite significant needs to monitor and anticipate the many epidemics emerging in isolated and marginal regions. Using mobile technologies for public health purposes (mHealth), it is possible to collect data and monitor health phenomena across time and space [4]. Such technologies rely on the availability of a far-reaching telephone network and free digital platforms that enable simple, rapid, and accessible implementation of a geo-referenced health surveillance system [5].

Among non-infectious ‘epidemics’ also requiring surveillance are road traffic injuries [6,7], which are major health events. These are a primary cause of mortality and morbidity globally, and particularly in LICs; in Africa they are the fifth leading cause of mortality [8]. Their consequences have impacts not only on population health, but also on countries’ economies and societies. It has been estimated that the direct costs of road traffic injuries represent 3% of gross national product globally and up to 5% in LICs [7,8]. It is therefore important that traffic injuries be tracked more carefully as a first step in identifying strategies to alleviate this burden.

However, in most African LICs, it is very difficult to assess the exact number of road crashes, where they occur, and their mortality/morbidity consequences. The lack of trauma data in these countries has long been recognized and deplored [3,9], but little has been done to remedy the situation. The United Nations’ Decade of Action for Road Safety [10] has prompted action in many regions, but almost none in Africa.

In Burkina Faso, the issue of road crashes and traumas has not been given priority in public health strategies, mainly due to a lack of resources and the profusion of other priorities requiring attention [11]. Yet the country and particularly its capital city, Ouagadougou, are much afflicted by this burden. Media reports of fatal crashes are numerous and increasing in frequency [12]. Occasional surveys have been conducted, but there is no ongoing surveillance system to provide much-needed evidence on which to base targeted actions that could reduce such events [13].

Burkina Faso’s National Police tally the number of collisions on public roads from statements by officers at the scene. The National Firefighters’ Brigade (BNSP) looks after the majority (70%) of those injured in road crashes [11]. It tracks the number of persons treated for injuries sustained in road crashes and produces annual mortality and morbidity reports. However, the police and BNSP reports never coincide. For instance, in Ouagadougou, for 2014, the BNSP reported 7818 road crashes and 152 deaths, whereas the police counted 13,173 road crashes and 145 deaths. In both organizations there are biases and under-reporting with regard to data collection and entry that explain, to some extent, these numbers and the poor reliability of the data. This discrepancy between police reports and those of other rescue and treatment services occurs especially often in low- and middle-income countries [14]. While solutions involving capture–recapture methodologies [15,16] can be applied to the police and hospital databases to estimate the number of deaths and injuries, even those have significant limitations in certain countries such as Burkina Faso. For example, neither the National Police nor the Yalgado Ouédraogo Hospital have computerized databases on road traffic crashes and injuries.

As such, there is a need in Burkina Faso, and in other LICs, to develop tools for reporting road crashes and traumatic injuries so that evidence can be produced to better understand this phenomenon that has become a major problem. The use of mobile technologies and social media has already been shown to be effective in public health surveillance [17], although this was in middle-income countries, such as South Africa. The challenge in LICs like Burkina Faso is to develop a tool that is easy for officers to use, low-cost, effective, durable, and designed in such a way that the knowledge produced is geared toward both users and decision-makers.

The objective of this article is to present the methodology and implementation of, and quality of results produced by, a prototype of a road traffic crash and trauma surveillance system implemented in partnership with the National Police in the city of Ouagadougou.

## Methods

### Study context

Burkina Faso, a landlocked South-Sahelian country in West Africa, is one of the 10 least-developed countries in the world. Its capital, Ouagadougou, is a city of two million inhabitants that is undergoing rapid urbanization and major demographic, health, and social changes. Its growth has been poorly controlled and it remains a very heterogeneous urban conglomeration, combining a modern urban center with dense, unsanitary residential areas and irregular urban boundaries.

The surveillance system was deployed over a six-month period, from February to July 2015, across the entire city. All seven patrol units of the public road crash intervention service of the National Police of the Centre Region collaborated with the research team.

### Data collection tools

The data collection tool implemented in this study was a prototype of a road crash epidemic surveillance and spatial-temporal monitoring system developed using Ushahidi, an open-source tool that applies the crowdsourcing concept to mapping and geographic information. Ushahidi (‘witness’ in Swahili) uses SwiftRiver, a free open-source platform that allows information to be extracted very rapidly and then restored after being filtered and verified. Sources include a variety of channels such as Twitter, SMS (short message service), email, and Really Simple Syndication.

**Figure 1.**
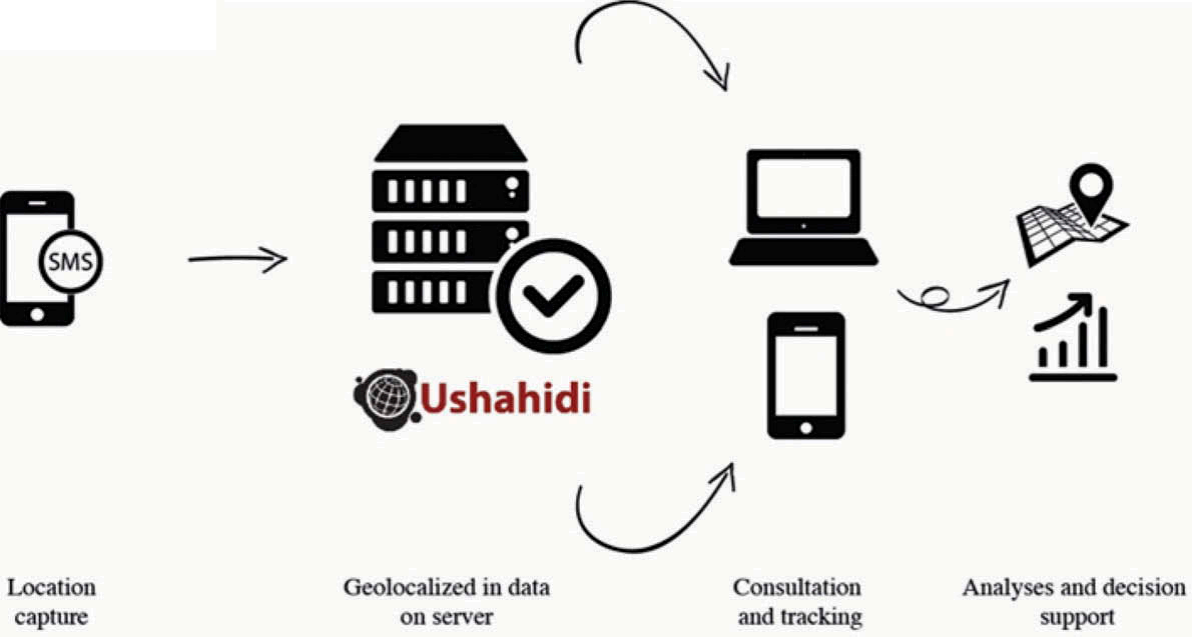
Collection and analysis of data from the road traffic and traumatic injuries surveillance system. Source: Bonnet (2016).

The road traffic crash surveillance system was based on the use of geotracers – GPS (global positioning system) devices with a telephone chip that enables the geotracer’s position to be transmitted by SMS. Other data collection solutions would have been possible using smartphones and a dedicated data-capture application, but the National Police administration, concerned that smartphones would be lost, rejected this option. A back-end application was developed to handle the GPS devices’ SMSs and link them with the Ushahidi mapping system. This was activated by a police officer at the crash scene. Data were sent in real-time to the platform, which analyzed the SMS content and extracted the relevant information (geographic coordinates) to integrate it into the Ushahidi interface within the map. The capability to detect and remove duplicate reports of the same crash or vulnerable road user (pedestrian or cyclist) collision was also added. The platform was Internet-accessible (http://traumatismes.africasys.com/) for consultation and data exportation (Figure 1). To complete the information on the event, data regarding sex, age, type(s) of vehicle(s), injuries, and deaths among the populations involved were collected on paper from the reports prepared by the officers. These data were entered daily into a database.

The core systems ran on a Linux server powered by Ubuntu 12.04 LTS with 4 Gb of RAM, 2 CPU and 20 Gb of disk space. This main server ran all the applications (Ushahidi, database system, SMS management system, incidents filtering/duplication removal, web interface, etc.).

The platform’s data (files and databases) were synchronized automatically at 2:00 a.m. daily on another backup server (which had the same hardware specifications as the master server). With this architecture, it was possible to restore backups in case of corruption and also switch to the backup server in case of outage to minimize platform downtime.

Twenty-one National Police officers were trained in the use and maintenance of the geotracer during a one-day training session. Refresher sessions were conducted during monthly visits to police stations. To ensure continual functionality of the geotracers, systems were installed so that they could be charged from the batteries of the police vehicles.

### Spatial analysis

The surveillance platform allowed the cases (crashes) that were geolocated and aggregated on the map to be exported to the geographic information system (GIS) and other statistical software. Spatial analyses (kernel density estimator) showed concentrations of traffic crashes in the city. Data analysis was performed using ArcGIS 10.3 for spatial analysis.

## Results

### Surveillance system feasibility: a perfectly functional platform

The process of data collection by police officers using geotracers functioned well. Researchers were able to validate the data collection on crashes by comparing the number of entries in the platform against the number of reports completed by the crash intervention teams. The implementation protocol for the system was based on continuous communication between the research team and the National Police stations. This cooperation enabled problems, such as geotracer malfunctions or improper handling, to be resolved as soon as they arose. However, putting the seven geotracers in place and synchronizing the data collection took about a month. Supplementary configurations of SMS messages were needed, as well as refresher sessions with officers regarding equipment handling and maintenance. Other equipment-related problems appeared near the end of the six-month period. Several geotracers malfunctioned, mainly due to lack of protection from the heat of the vehicles in which they were installed.

Table 1 presents the total cost of the surveillance system over three months, without counting the research activities.

**Table 1.** Surveillance system costs over 3 months, excluding research activities.

Expenses	Unit cost (Euros)	Quantity	Total cost, 3 months (Euros)
Geotracers	152	7	1064
S.M.S	0.015	873 x 2 (confirmatory messages between platform and geotracer)	26
Server hosting	1800	1	1800
Server hosting per year as of N + 1	1300*		0
Installation/backup	1200	1	1200
Support interventions	600	1	600
Total			4690*

*Total cost for installation and three months of surveillance. Total annual cost after year 1 is estimated at 1350 Euros.

Both the methodology and the implementation of this simple surveillance system were nevertheless satisfactory and the results over a period of three months during which the process functioned perfectly illustrated the system’s knowledge-producing capacity. The SMS-based system for disseminating crash data validated the proposed process, because it could be replaced by a simple telephone equipped with geolocalization.

**Figure 2.**
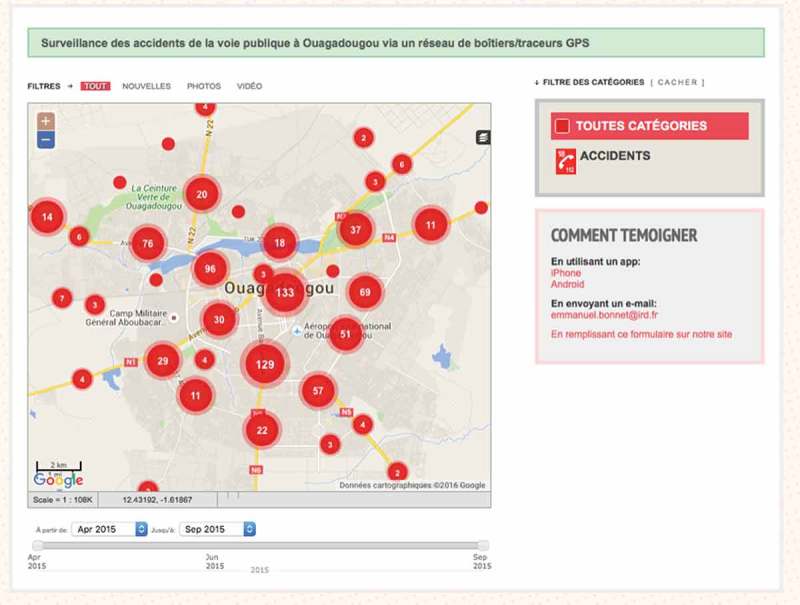
Screenshot of the Ushahidi interface for the Ouagadougou road crash surveillance system.

**Figure 3.**
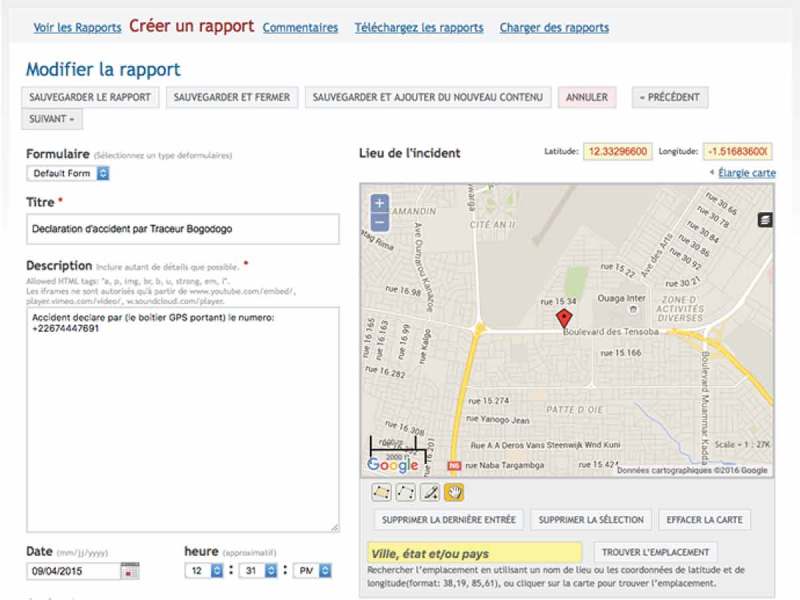
Geolocalized visualization of a crash.

**Figure 4.**
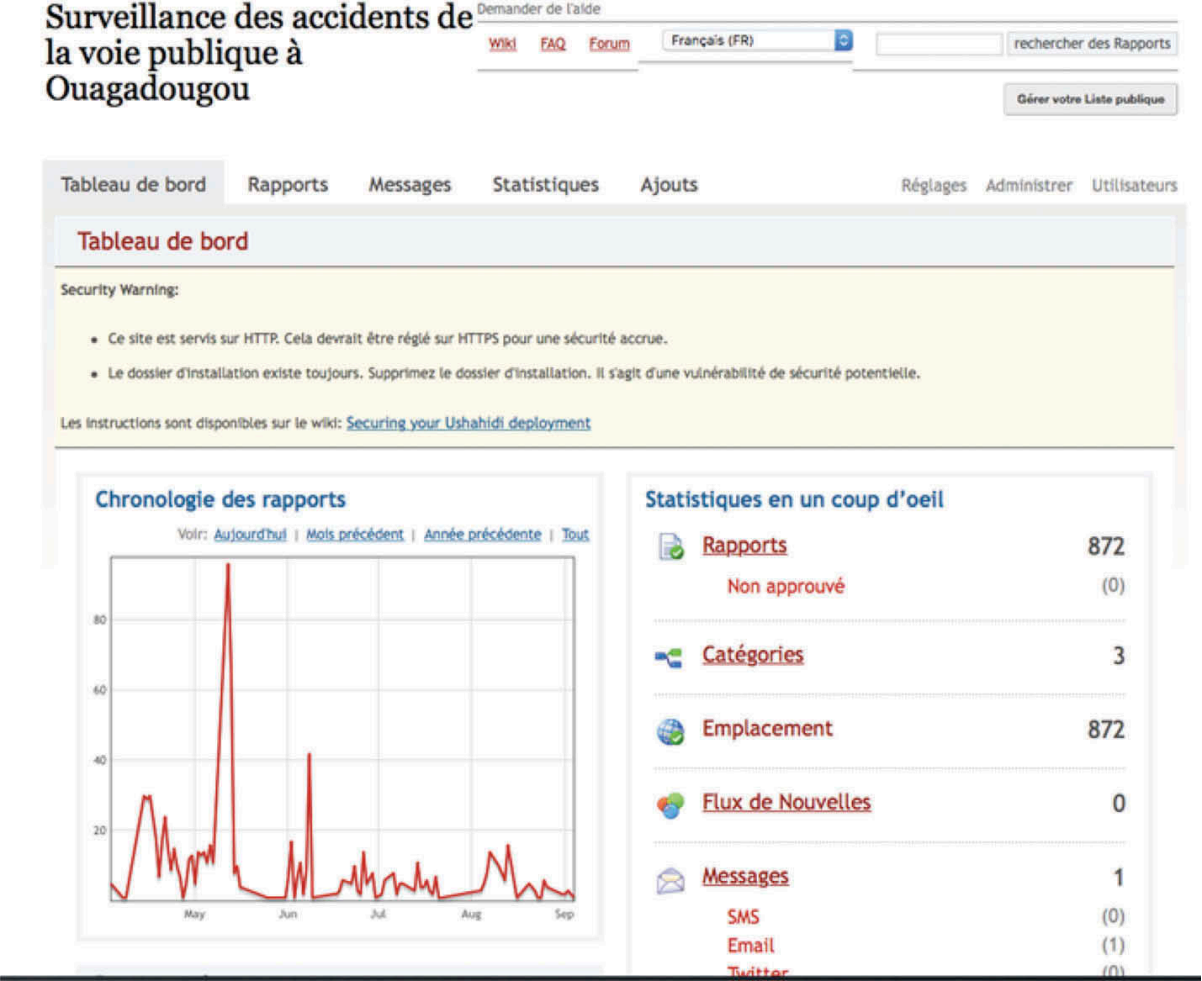
Temporal monitoring of a crash via the interface.

In total, 873 crash scenes were recorded over 3 months. The system was accessible on the Internet (Figure 2) for open consultation of the map of crash sites, with restricted access to data on precise locations and time-related information. With the map interface it is possible to zoom in and observe the geographic distribution of crashes in a district, and even, for example, on a specific street (Figure 3). The interface also makes it possible to monitor crashes over a defined period to assess fluctuations and carry out temporal interpretations of the distribution of crashes (Figure 4). All data acquired and integrated into the database could be exported, with no particular conversion problems, from the platform into statistical or spatial analysis software. Descriptive statistical analyses revealed that crashes occurred more frequently in the evenings and during rush hours, as well as on weekends and Mondays. They occurred primarily at intersections with traffic lights. With regard to health impacts, half of the injured were under the age of 29 years, and 6 persons were killed.

The system could be accessed via the Internet everywhere in the city of Ouagadougou, including in 3G. While police officers did not use this function, testing by the research team showed the feasibility of accessing the surveillance system.

### Quality of data produced

**Figure 5.**
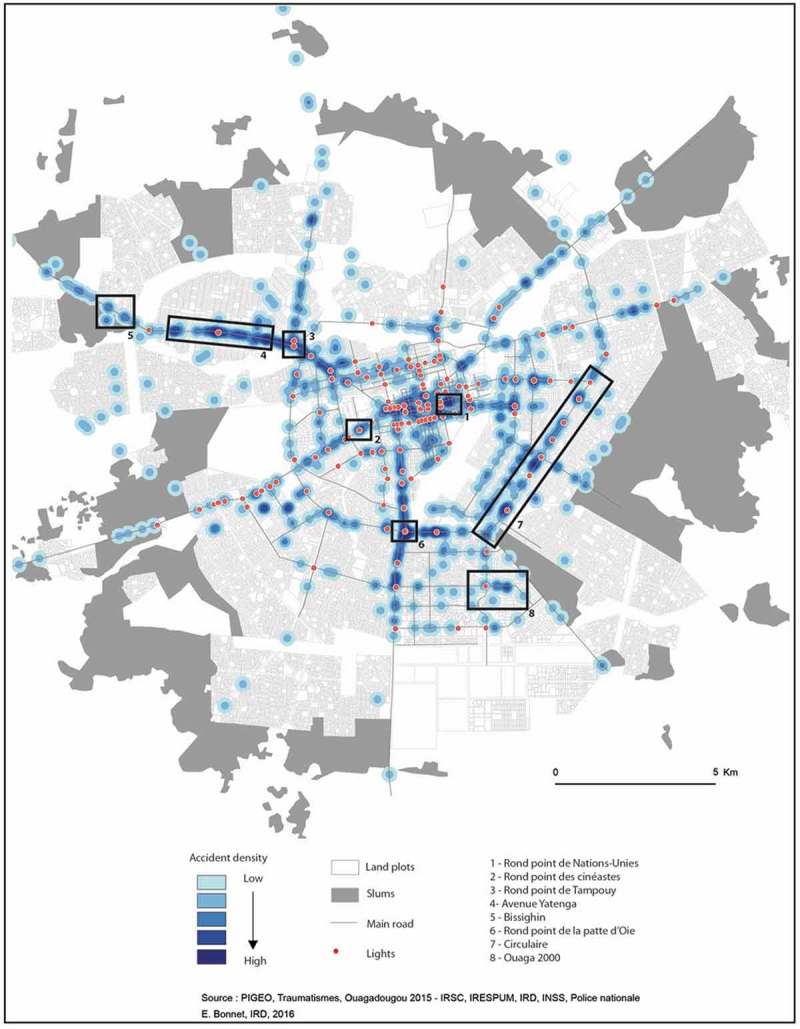
Map of crash ‘hot spots’ in Ouagadougou, April–June 2015. Source: P.I.G.E.O, Traumatismes, Ouagadougou 2015 – I.R.S.C, I.R.E.S.P.U.M, I.R.D, I.N.S.S, Police nationale. E. Bonnet, I.R.D, 2016.

To illustrate the system’s interoperability and quality, a map was created of the ‘hot spots’ of crashes in Ouagadougou (Figure 5) by exporting the geographic locations of the crashes. This exportation toward the analysis tools, carried out with no conversion or data cleaning problems, demonstrated the quality of the data produced and archived by the surveillance system. This allowed phenomena to be analyzed from different perspectives, which is essential for implementing prevention measures.

Without entering into the details of future articles that will analyze these data in greater depth, here we point out the mapping of ‘hot spots’ that highlighted several concentrations of crashes on the paved roads of the capital. Two main hot spots appeared in the city center: one at the United Nations roundabout (area 1 in Figure 5), where the city’s main arteries converge, and one at the Film-makers’ (*cinéastes*) roundabout (2), another convergence from the center toward the main highways of the capital. The next largest concentrations were observed at the Tampuy roundabout (3) and then at Yatenga Avenue (4), an area that is becoming increasingly densely inhabited. This densification has led to the development of activities all along the roads, related particularly to the presence of unauthorized residential districts, such as that of Bissighin (5). Two other concentrations of crashes were observed at the junction roundabout (6) and the circle (7). Again, these correspond to points of convergence and bypasses around the central districts, or to roads leading to new administrative poles, such as the Ouaga 2000 district (8).

Analysis of these concentrations showed crashes on the city’s main arteries, in areas of convergence. However, significant concentrations were also observed on recently paved roads not yet equipped with traffic calming or other features, nor even with traffic lights. Likewise, hot spots were observed near traffic lights that were poorly respected by drivers. District maps were produced for the police stations in the different zones so they could analyze their territories and deploy officers to the crash-prone locations.

## Discussion

The burden of road injuries is significant in most LICs, and Burkina Faso is no exception. Similarities can be observed with other countries, such as in Asia, in terms of under-reporting of crashes and of numbers of victims. In Pakistan, for example, a national inquiry was conducted to prevent such under-reporting and to assess the increase in traumatic injuries associated with traffic crashes [18]. In Africa, as elsewhere among Southern countries, systematic data collection is essential to assess the real burden of traffic crashes [19,20].

The surveillance system prototype developed and tested in a real-life context over a six-month period in Ouagadougou produced high-quality results, not only from a methodological standpoint, but also in terms of implementation feasibility and results obtained. In fact, the mapping of hot spots supplemented a survey conducted by the emergency trauma services of Yalgado Ouédraogo Hospital in Ouagadougou. Conducted over the same period, it provided information on the epidemiology of traffic traumas. It counted 1867 victims admitted to the main hospital and 47 deaths. Of the injured, 87% had been travelling on two-wheeled vehicles (e.g. bicycles or motorbikes). More than 50% of the injured were under the age of 29 years [11]. Nearly 45% of injuries were head traumas, and 26% of the victims continued to present with disability 1 month post-event. Lastly, the average cost of treatment per patient was 126,799 CFA francs (230 CAD) [11], nearly four times the average monthly salary in Burkina Faso. Other, more analytical articles on the epidemiological aspects of this study are currently in process. One objective of this article is to first present an innovative methodology and data collection process.

These different data collection systems are separate elements that provide the foundations for a trauma surveillance system. They could easily be implemented with the support of everyone working on road safety in Burkina Faso. Authorities responsible for transportation, interior security, and health recently reacted positively to the results presented at the First African Road Safety Forum (FASeR) (http://faser-afrosafe.org) in May 2015 and at the workshop to present the findings of this study in November 2015 (http://www.equitesante.org/equiteburkina/axe-recherche-traumatisme/). A deliberative workshop conducted with all those involved in road safety was effective in engaging the collaboration of the community as well as non-governmental and governmental organizations in monitoring, preventing, and reducing traffic crashes in Burkina Faso. The goal now is to develop a territory-wide surveillance system that will track crashes, treatment, and follow-up of patients throughout their recovery process. It is imperative that the authorities be committed to implementing this system, because the National Police hierarchy is the key to having it accepted by officers on the ground. That commitment is not obvious in Burkina Faso, nor in other LICs, where controlling and preventing traumatic injuries is not a priority for politicians [21].

Presenting and gaining acceptance for the implementation of this trial project took approximately one year, because officers had to be persuaded to add another task to their daily activities. This had to be done without disrupting their routines, and without offering any compensation or administrative premium, which are the norm in aid-dependent countries [22]. Even though there were times when they were less motivated to use and maintain the geotracers, continuous communication between the authors’ team and the National Police officers helped overcome any lapses. The presentation of results during our regular visits with the patrol units of the National Police, and at the end-of-project workshop, convinced the officers and their superiors of the value of such a system. This clearly demonstrates the importance of ongoing and transparent sharing of research results with decision-makers, which is still too rarely done in Africa [23]. For deployment on a larger scale, therefore, it will be important to plan for significant periods of negotiation with the authorities. These negotiations to illustrate the magnitude of the situation with regard to road crashes and traumatic injuries are difficult to implement [24]. Yet they are indispensable for ensuring orders are issued and for validating the line ministries’ involvement in the study and its intervention. This is even more important now, given that, since the election of the current President of the Republic in 2015, the Ministry of Transport has extended its authority to urban transit and road safety.

These surveillance systems and their associated technologies have been shown to be effective, simple, and affordable. In a context where there is a significant paper-oriented culture, incorporating the systematic use of these tools into police surveillance practices is a challenge. National Police officers agreed to use these new tools, and we invited them to the results presentation to include them in the analysis process and to solicit their advice on the technology and how it might be adapted. One idea that emerged from this was the potential development of a data collection tool that would use a smartphone with a dedicated application, rather than a geotracer, especially because it would then be possible to enter into the form items related to the officer’s report and to the people involved. These data, theoretically compiled in the BAAC (*bulletin analyse des accidents corporels* – physical injuries analysis report) file, could be compiled directly into the Ushahidi platform. Moreover, using smartphones would avoid the functional problems encountered with the use of geotracers. One of the positive points of this experience was the low cost of this surveillance system. The devices for sending the data, whether geotracers or smartphones, are affordable, at around 150 Euros. Also, sending SMSs is not difficult. The platform was developed using very effective open-source tools. The only costs – for development and maintenance – were incurred at the beginning of the deployment, but these were considered low in relation to the value of a good-quality system for surveillance of traffic crashes and their health consequences.

Besides GPS-equipped smartphones, other elements are needed to deploy these technological solutions, such as a remote computer server that will ensure continuity of data acquisition and can cope with power interruptions – a frequent occurrence in Burkina Faso – and training for health workers and surveyors in the use of the data collection tool to make them aware of the time-saving aspects, reliability, and value of surveillance systems.

Implementing electronic surveillance systems will support public health, and these innovative tools will provide benefits not only for the health system, but also for all parties involved in the road safety sector. The overall contribution of electronic health records has been widely demonstrated globally, and their application in the South is becoming equally relevant, as shown by our experience in Ouagadougou [25]. If this data collection system were to be implemented for the National Police and the hospital, regular and comprehensive statistics could be produced without having to resort to statistical estimation methods. In particular, this would make it possible to capture data on crash victims who go on their own to the emergency room without waiting for the police to arrive at the crash scene. Conversely, those with minor injuries who go to health centers for treatment after the police intervention could also be entered into the system. While many studies have used capture–recapture methodologies to estimate such unreported cases [15], all have stressed the need for data collection systems to be improved [26] to minimize discrepancies in the victim counts. Estimation methodologies are effective *a posteriori* to assess the burden of crashes; still, they are imprecise and often far from accurate. Only synchronized data collection by all parties involved (police, firefighters, and hospitals) can produce accurate counts of the numbers of crashes, deaths, and injuries.

However, the present study did have certain limitations. There was uncertainty about whether the data were exhaustive. In fact, the data collection carried out by the National Police matched the crashes to which they were summoned. In Burkina Faso, collision reports are prepared by the police and incur fees (3000 CFA francs = 4.6 Euros); these reports are required for insurance coverage when there are material damages. Likewise, the National Police intervene systematically whenever there are persons injured in a crash. Consequently, collisions without serious injuries are not counted, being resolved privately by the parties involved. The data collected in this study also did not take into account individuals who were injured but went to the emergency room on their own. In the study conducted by the hospital, 15% of those injured in traffic collisions [27] came to the emergency room by their own means. However, these study limitations apply equally to paper-based data collection by officers at the scene, to which are added significant delays in data entry, human error, and occasional loss of original documents. Thus, while this surveillance system has certain limitations, they are no different from those of the paper data collection routinely used.

## Conclusions

This pilot study demonstrated the feasibility of developing simple surveillance systems, based on mHealth, in LICs. The system’s acceptance by police officers and authorities was a major outcome of this study. While this acceptance could not be directly measured, the officers’ level of use of geotracers on public roads provided indirect evidence. Other studies have shown the suitability of mobile telephone use in the South [28], but none has yet explored its use for mapping purposes in the context of crash analysis and road injuries.

The principle behind any public health surveillance system is to comprehend the extent to which health problems are occurring. The basic aims are to: detect occurrences and their distribution; monitor long-term trends and causes of disease; and identify changes and developments in practices [2]. From this standpoint, the platform tested is incomplete. The challenge is to enable these systems to function interactively with other healthcare recording systems (currently based on paper registers), or to develop them with emergency services departments, as well as to scale them up nationally, as the burden of road traffic crashes appears to be greater outside of urban areas. Regardless, the road traffic crash surveillance system we created is already relevant in terms of road safety, upstream from health issues. For instance, this system enabled police forces in Ouagadougou to intensify their presence at ‘hot spots’ and to engage in control and deterrence activities. Even so, this system is only a first step before implementing a trauma surveillance system that would track crashes, traumatic injuries, and long-term consequences for victims. There is currently no such comprehensive road safety program in West Africa, nor in most LICs. It is essential that such systems be implemented widely in LICs to contend with a phenomenon that should, in today’s world, be considered a public health priority not only in Africa, but in all Southern countries [25].

## Paper context

Data on crashes and related injuries are scarce in many low-resource settings despite the huge associated burden of morbidity and mortality. Capture–recapture statistical analysis is one method of estimating the incidence of road traffic deaths and morbidity that bypasses the limitations of incomplete data sources. Meanwhile, advances in freely accessible and easy-to-use information and communications technology can contribute to public health data collection. An experience is presented in Burkina Faso that could be extended to other Low and/or Middle Income Countries.
